# Evaluation of Consumers Perspective on the Consumption of Antibiotics, Antibiotic Resistance, and Recommendations to Improve the Rational use of Antibiotics: An Exploratory Qualitative Study From Post-Conflicted Region of Pakistan

**DOI:** 10.3389/fphar.2022.881243

**Published:** 2022-05-18

**Authors:** Faiz Ullah Khan, Tauqeer Hussain Mallhi, Farman Ullah Khan, Khezar Hayat, Asim.Ur Rehman, Shahid Shah, Zakir Khan, Yusra Habib Khan, Tawseef Ahmad, Sai Krishna Gudi, Yusuf Karataş, Yu Fang

**Affiliations:** ^1^ Department of Pharmacy Administration and Clinical Pharmacy, School of Pharmacy, Xi’an Jiaotong University, Xi’an, China; ^2^ Center for Drug Safety and Policy Research, Xi’an Jiaotong University, Xi’an, China; ^3^ Shaanxi Center for Health Reform and Development Research, Xi’an, China; ^4^ Research Institute for Drug Safety and Monitoring, Institute of Pharmaceutical Science and Technology, Western China Science and Technology Innovation Harbor, Xi’an, China; ^5^ Department of Clinical Pharmacy, College of Pharmacy, Jouf University, Sakaka, Saudi Arabia; ^6^ Institute of Pharmaceutical Sciences, the University of Veterinary and Animal Sciences, Lahore, Pakistan; ^7^ Department of Pharmacy, Quaid-i-Azam University, Islamabad, Pakistan; ^8^ Department of Pharmacy Practice, Faculty of Pharmaceutical Sciences, Government College University, Faisalabad, Pakistan; ^9^ Institute of Health Sciences, Department of Pharmacology, Faculty of Medicine, Cukurova University, Adana, Turkey; ^10^ Pharmacovigilance Specialist, Balcali Hospital, Faculty of Medicines, Cukurova University, Adana, Turkey; ^11^ Department of Pharmacy, COMSATS University Islamabad, Abbottabad Campus, Abbottabad, Pakistan; ^12^ College of Pharmacy, Rady Faculty of Health Sciences, University of Manitoba, Winnipeg, MB, Canada

**Keywords:** customers, antibiotic use, antibiotic resistance, conflict, community pharmacy

## Abstract

**Background:** Antibiotics misuse is a global challenge, and the situation is likely to deteriorate in conflict zones with insufficient health services. The misuse of antibiotics is not only associated with antimicrobial resistance but may also lead to serious consequences. This study was aimed to investigate the knowledge, attitude, and practices on antibiotic consumption, antibiotic resistance (ABR), and related suggestions among residents of conflicted zones in Pakistan.

**Methods:** Semi-structured interviews were conducted at community pharmacies between June 2020 and January 2021. The primary findings were ascertained through thematic content analysis. Themes, sub-themes, and categories were drawn from the final analysis. Data analysis was carried out in six steps from getting to know the data to final report development.

**Results:** A total of 20 consumers were interviewed with a mean interview duration of 25.4 min. The average age of participants was 35.1 years, and most of them were males. ABR was unfamiliar to the participants. Most of the participants understood the term “antibiotics,” but they did not know how to use them properly. The participants were unable to distinguish between bacterial and viral illnesses. Thirteen participants believed that antibiotics have a faster effect than any other drug. Most of the participants perceived that every antibiotic could cause diarrhea, and pharmacy staff sometimes prefer other medicines such as multivitamins. Consumer practices regarding antibiotic usage and ABR were found to be poor. Most participants recommended that health officials must ensure qualified staff at pharmacies with strict regulations. Five participants said that a leaflet with antibiotic instructions in Urdu (national language) is usually beneficial, especially when making solutions from powder.

**Conclusions:** This study underscored poor knowledge, attitude, and practices among residents of conflicted zones towards antibiotics and ABR. Low literacy rate, unavailability of healthcare facilities, absence of pharmacists at community pharmacies, and uncontrolled sales of antibiotics are some factors attributed to serious hazards, ABR, and irrational use of drugs.

## Introduction

Antibiotics are considered as life-saving drugs; although the 19th century was dubbed the “Golden Era” as a result of its discovery, the golden period did not last long due to the rise of various resistant infections (Akhund et al., 2019). ABR is a long-standing problem (Khan and Fang, 2021). ABR has drastically expanded in the twenty-first century and is primarily attributed to irrational prescription and incorrect usage. It has become a major public health hazard worldwide, especially in underdeveloped countries (Laxminarayan et al., 2013). Antibiotics are among the list of most prescribed medications, thereby possessing a substantial risk of drug abuse. According to an estimate, approximately 20–50% of antibiotics prescribed to patients are inappropriate (Ahmed et al., 2020). The poor policies for infection control, under/excessive use of antibiotics, and availability of over-the-counter antibiotics are some of the major drivers of ABR (García et al., 2011).

Between 2000 and 2015, the global use of antibiotics increased by 65%, primarily driven by increased consumption among low- and middle-income countries (LMICs) (Klein et al., 2018). Among LMICs, the highest consumption of antibiotics was observed in India, China, and Pakistan in 2015. Of particular note, antibiotic consumption increased by 65% (up to 1.3 billion Daily Defined Doses) in Pakistan in 2015 (Atif et al., 2021). If timely measures are not taken, it is estimated that ABR might cost 10 million deaths by 2050 (DeBaun et al., 2021). On the other hand, inappropriate use of antibiotics adversely affects patients by increasing the length of hospital stay with out-of-pocket expenses, which eventually diminishes the health-related quality of life (Atif et al., 2020).

It has been observed that knowledge and awareness of ABR are considerably low in countries having an increased burden of antibiotic resistance cases (Grigoryan et al., 2007). As a result, public information campaigns are launched in various countries as part of a national strategy with diverse levels of effect on the antibiotic cogent usage (Cross et al., 2017). ABR has assessed its substantial burden in Europe at €1.5 billion each year (Organization, 2018). Similarly, in the United Kingdom, one out of every four antibiotic prescriptions is erroneously filled, resulting in 10 million improper prescriptions each year (Mason et al., 2018). Understanding consumers’ knowledge of antibiotics and their appropriate use is vital in identifying the public’s attitude that consequently aids in shaping the campaigns and policies on this public health issue. The World Health Organization (WHO) has proposed public education as one of the key interventions to rationalize the use of antibiotics (Chang et al., 2017). Enhancing the public understanding of antibiotics is the principal strategy of the WHO`s Antimicrobial Resistance Global Action Plan (the World Health Organization, 2015).

The available body of evidence from Pakistan indicates that the use of antibiotics is still inappropriate in spite of various antimicrobial stewardship programs (Khan et al., 2021a; Khan et al., 2021b). Evidence suggests that the general public plays an essential role in curbing the growing encumbrance of ABR (Khan et al., 2020). Moreover, consumers’ behaviors towards antibiotic use are of paramount importance in post-conflicted areas with poor healthcare facilities.

Due to the heavy military operations (2007–2011), infectious diseases are on the rise in the district, where respiratory tract infections are more common. Unfortunately, schools, hospitals, and roads are still destructed and have not been rebuilt even after a decade of war (Saeed Khan, 2019). The prevalence burden of infectious diseases might be attributed to the displacement and evacuation of citizens to the other parts of Pakistan where infections are more prevalent (Muzamil et al., 2021). The war in the Swat district has affected people of every walk of life and still infectious diseases are most prevalent, and this is relevant with the current study to measure the knowledge, attitude, and practices toward antibiotics and ABR.

The findings of the current study would help the policymakers to better understand the consumer perspectives toward ABR, which could be translated into appropriate strategies for strengthening the healthcare system in Pakistan.

## Methods

### Study Setting

A qualitative research (semi-structured interviews) study was carried out in post-conflict areas, formally known as the “Yousufzai state of Swat (1849–1969).” The current population of Swat is 2.3 million, and it is one of the rural districts in the northern region of the Khyber Pakhtunkhwa Province, Pakistan. The majority of the people live in rural areas, comprising about 86% of the state. Swat has witnessed an extensive militancy conflict since 2007. In this study, mainly Swat district was targeted for the data collection from June 2020 to January 2021. An explorative phenomenological study design was used, based on the face-to-face interviews.

### Study Design

This was a qualitative study that employed semi-structured interviews to uncover participants’ perceptions about antibiotic usage and antibiotic resistance along with recommendations. Semi-structural interviews are useful tools, especially for exploratory studies. The study design offers several advantages such as the ability and flexibility to thoroughly examine the knowledge, experiences, and purpose of the participants on a specific subject. The interview schema contained several questions with sub-sections. In a profound conversation (face-to-face in-depth interviews) with the consumers, their general perceptions of antibiotics and ABR were assumed to determine. The interviews had been designed and conducted at the site of each pharmacy, and a place was reserved in the waiting area to ensure complete easiness for interviewers. All interviews were carried out in the Pashto language for the given population’s convenience. It took approximately 20–30 (minutes) to complete each interview. For recording interviews, a voice recorder was utilized, and the recording had been kept and saved confidentially. The pharmacies situated in the post-conflicted/war-affected areas were selected randomly.

### Inclusion Criteria

Consumers were eligible to participate in the study if they met predesigned criteria. The first was that all participants in the offered pharmacies had to be over the age of 18 years and had to have been invited on a pure volunteer basis. Second, we had approached consumers who had made their visit to selected pharmacies and made a demand for antibiotics, whether they had a prescription or not or had empty packings or strips of antibiotics. A separate space in the selected pharmacies was reserved for the participants to feel easy during the interview. Participants also had to be able to read and understand Urdu/Pashto (national/local language) and give informed consent orally or in writing. Consumers who had no physical or mental disability were included in the study. Participants who did not meet the inclusion requirements or who refused to participate in the study were excluded.

### Development of Interview Guide

**FIGURE 1:**
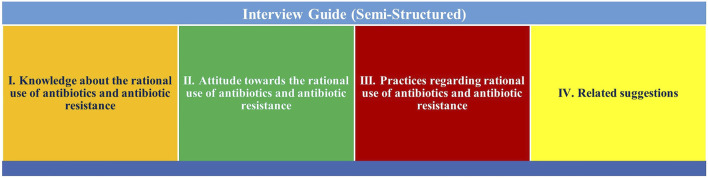
In-depth semi-structured interview guide summary of the themes.

An interview guide was developed through an extensive literature survey based on a qualitative and explorative study design (Côté and Turgeon, 2005; Norris et al., 2013; Ancillotti et al., 2018). The final version of the semi-structured interview guide was approved by two experts and translated by a skilled academician to the local (Pashto) language. A minor revision was carried out as per the direction of the pharmacy experts. The principal investigator’s first author had to compare the translated version with the original English one and achieve equivalence (Hendrickson et al., 2013). Pilot interviews were undertaken on three interviewees for the test of protocol for confirmation of understandability and validity. However, the pilot interviews were not included in the final study. The interview guide used in the current study is provided as Supplementary File S1. The summary of the topics for the in-depth interviews is shown in Figure 1. General population views about antibiotic resistance matter to know the literature gap.

**FIGURE 2:**
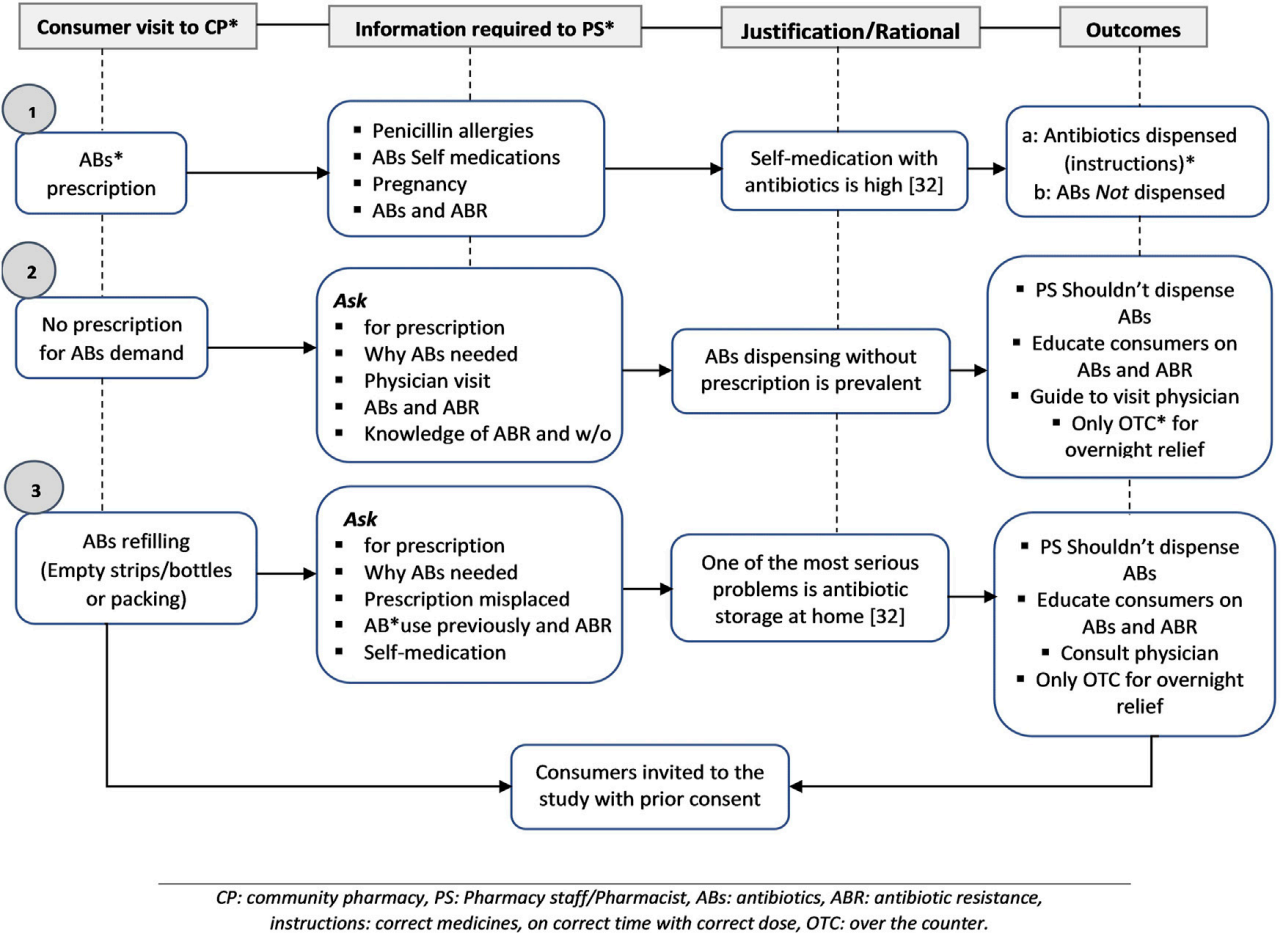
Three scenarios for the consumer’s approach to the selected community pharmacy.

Three major scenarios were established before the study. The first option was for the consumer/patient to visit the selected pharmacy with a prescription that includes antibiotics. The second option was that the consumers did not present doctor prescriptions and demanding for antibiotics. The last scenario was about the patient visiting a pharmacy to refill antibiotics without a prescription having empty strips/bottles or other medicine packaging. All the detailed scenarios with description and pharmacy staff approach are shown in Figure 2 with rationale.

### Sampling Technique

Purposive sampling is considered a basic technique of sampling and was used in the present qualitative research (Etikan et al., 2016). Moreover, the given techniques that were helpful in the representative sample and range of understandings could be obtained effectively to show the variety in the sampling (Côté and Turgeon, 2005; Etikan et al., 2016). The inclusion of the participants was purely based on their locality, age, education, and economical status. The principal investigator (FUK) visited all the selected data collection points of the community pharmacies.

The data collectors (pharmacists) were aware of the given regions and locality. The interviewers provided all the information orally and in writing to the nominees. The principal investigator arranged the interviews for the consented participants. The in-depth interviews were initiated until gaining the point of saturation. A total of 20 participants were recruited for the study, and not a single participant was dropped from the study.

### Data Collection

Data were collected between September 2019 and January 2020. The criteria for saturation points had been used to establish the adequacy of the sample size. A two-stage approach was used to choose the participants. The first phase involved participants with antibiotic prescription who approached a pharmacy during working hours (9 a.m.–6 p.m.); identified and eligible participants were asked to join the study. The second stage was to choose and interview participants who agreed to engage in the study at a time and location that was pleasing to both sides. All interviews took place in Pashto. A qualified pharmacist carried out the semi-structured interviews. The transcripts and audio of the interviews were all transcribed. The participants of the study were able to read and hear taped interviews.

### Analysis of Data

The analysis was carried out utilizing Braun and Clarke’s thematic analytical approach (Braun and Clarke, 2006). Different steps were involved in data analytics processes from their thematic approach: knowledge of data, initial code development, thematic search, topic evaluation, identification and theme naming, and report development (Braun and Clarke, 2006). Initial data familiarization began when audio recordings were listened carefully, transcribed verbatim, and translated from Pashto to English. A forward and reverse transfer approach was tested on a group of transcripts for the translation process validation and was found to be reliable (Bowen, 2008). The initial codes for the study objectives were generated through open coding. To produce themes and sub-themes, the coded information was reduced. All authors examined the topics of the final theme. The emerging themes had been cross-checked and had been concluded through frequent research team meetings (Table 1). For the word cloud visualization and data codings, the qualitative data analysis software (NVIVO-19) package was utilized.

**TABLE 1:** Steps involved in data analysis.

Steps	Analysis	Accomplished task	Contribution of the research team	PI
Step-I	Getting to know the data	Recorded interviews	F.U.K (PI)	Reviewed the initials step
		Listening		
		Transcription		
		Reading and rereading		
Step-II	Creating the initial codes	Codes assign to data	F.U.K (Farman) and THM	Finalized the codes
		Further code initiation		
Step-III	Explore the themes	Classification of themes and into categories	F.U.K (PI) and S. S	Finalized the main themes
Step-IV	Themes critical review	Themes confirmation with the consistency	K.H, Z.K, and consultation with T. A	Reviewed the themes
Step-V	Identification with themes names	Themes are being refined even more	A.U.R confirmed with T. A	Final names assigned to the themes
Step-VI	Final report developed	Selection of the quotations	Y.H.K, F.U.K (PI) reviewed and confirmed by Y.F and Y.K	Final report confirmation

a PI, principal investigator.

## Results

**FIGURE 3:**
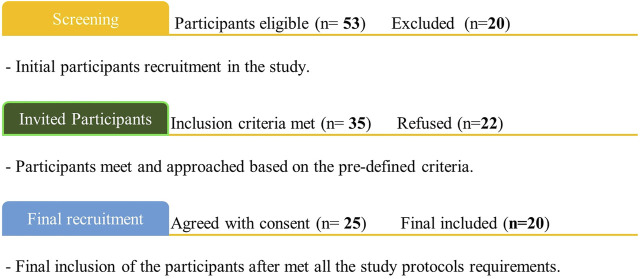
process of the participants’ recruitment.

A total of 20 in-depth interviews were conducted with a mean duration of 25.4 min. A detailed recruitment process from screening to final recruitment of the participants is described in Figure 3. Overall, *n* = 53 consumers were approached, 20 participants were excluded, and 22 refused to participate in the study. Finally, 20 participants were included in this study.

Most of the participants were males of age between 20 and 55 years (mean age of 35.1 years). The demographic parameters of study participants are described in Table 2.

**TABLE 2:** Respondents’ (gender, age, and interview duration) details.

Respondent	Gender^a^	Age (years)	Interview (duration min)
Respondent-A	M	31	25
Respondent-B	M	43	24
Respondent-C	M	55	27
Respondent-D	M	26	30
Respondent-E	M	29	20
Respondent-F	M	31	26
Respondent-G	M	25	30
Respondent-H	M	29	27
Respondent-I	M	32	21
Respondent-J	M	38	23
Respondent-K	M	40	26
Respondent-L	M	45	29
Respondent-M	M	23	30
Respondent-N	F	47	20
Respondent-O	M	28	31
Respondent-P	M	22	30
Respondent-Q	M	26	25
Respondent-R	M	52	20
Respondent-S	M	37	23
Respondent-T	F	39	21

a M, male; F, female

**FIGURE 4:**
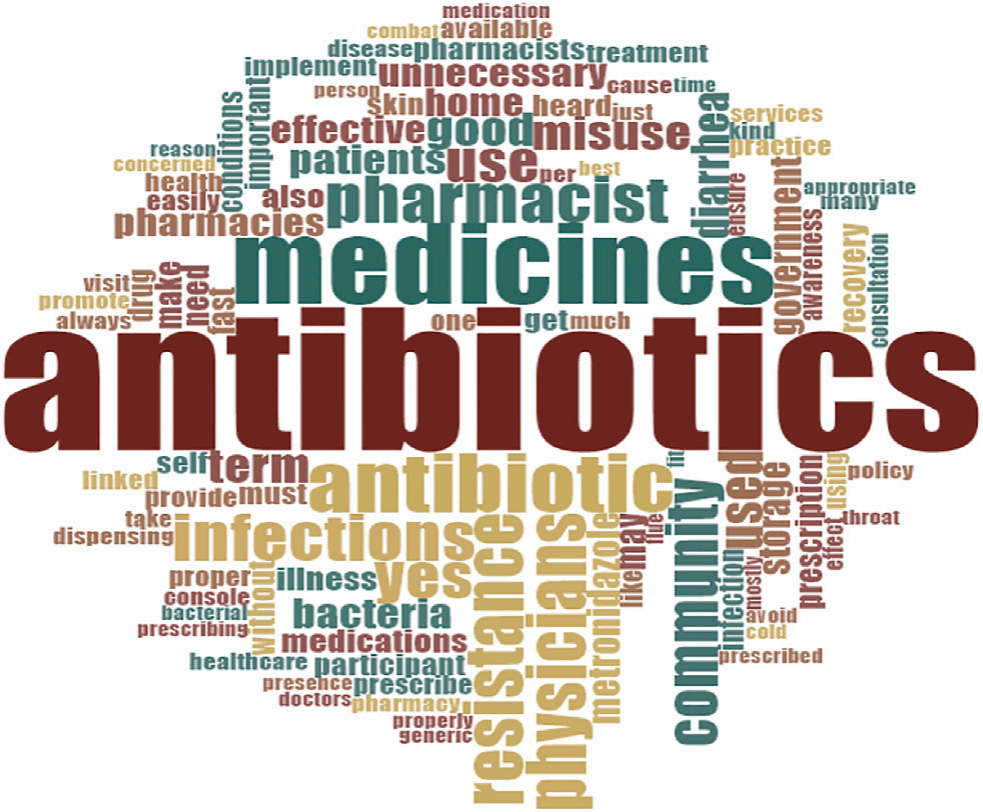
Word cloud diagram depicting the antibiotics use and the perceptions of the participants related to antibiotic use and antibiotic resistance.

Themes (4), sub-themes (6), and categories (31) were established, and the thematic analysis was applied. All themes included knowledge, attitude, practice, and rational use of antibiotics presented in detail. A cloud diagram was used to visualize the data as per the responses of the participants regarding antibiotics, ABR, and the perspective on the rules and regulations about the drug laws (Figure 4).

### Participants’ Knowledge

First, the participants’ level of knowledge of antibiotics and the extent to which they (interviewees) understood how antibiotics were used were determined. Basic knowledge questions related to antibiotics were asked. All the respondents at three pharmacies replied with a satisfactory answer and that they had heard the word ‘antibiotic.’ Most of the interviewees defined the ‘antibiotics’ correctly (Table 3).

**TABLE 3:** Participants’ knowledge related to antibiotics and ABR.

Theme 1: knowledge related to antibiotics and antibiotic resistance		
**Sub-themes**	**Categories**	**Quotations**
Antibiotics (general) knowledge	Consumers somehow know the word antibiotics	Yes, I have heard the term antibiotics many times and mostly used when someone got injured or has wounds with peep (respondent-C)
	Believed antibiotics are always used in wounds and infections	For any type of disease (cold, fever, and flu) you can use antibiotics because of their work as per our experience and practices (respondent-M)
	The majority of the respondents were aware of common antibiotic names	The medicine erythromycin, which we use in our homes for throat illness or infection, is commonly available at drug stores (respondent-D)
	Virus and bacteria, confused recognition of the terms	Virus and bacteria are microorganisms and one can recommend antibiotics in such conditions which have referred to the virus or bacteria (respondent-B)
b) Knowledge of antibiotics resistance	Unaware of the term *“antibiotics resistance”*	Strange to know that resistance also exists in medicines, for the first time I heard ‘antibiotic resistance’ (respondent-A)
	Poor knowledge related to the terminology	I have heard the term antibiotics from my teacher, but I am unaware of antibiotic resistance and its mechanism (respondent-G)
	The actual cause is unknown	As per my understanding, antibiotics self-use is one of the reasons for antibiotic resistance (respondent-J)
	Wrong beliefs over misuse of antibiotics and resistance	I always use erythromycin for common cold and throat infections, as I recommend antibiotics to my family and friends (respondent-L)
		Antibiotics are the same drugs as ‘paracetamol’ we can get antibiotics easily (respondent-I)

The responders’ inability to explain when to take antibiotics and when to stop was the most eye-catching issue. As per their knowledge, “antibiotics should be administered if you have infections or any type of wound” without any confirmation that the infection-causing agent is a virus or bacterium (respondent-C).

Following basic questioning on antibiotics, queries on antibiotic usage as well as questions about ABR were asked. Overall knowledge about antibiotic resistance was poor, and after explaining the basic terms and definition of ABR to each participant, 9 out of 17 had heard the term ABR but ignored to ask from healthcare professionals. Only four respondents answered correctly, and two of them explained that ABR is a serious threat to global health.

Participants were unable to explain the exact factors behind, ABR but their knowledge about self-medication (antibiotics) was somehow satisfactory as one of the participants responded that “Many of us take antibiotics very easily without any hurdle” (respondent-J). Overall, interviewees had reported erythromycin as the most commonly used antibiotic. Self-recommendation (sharing of antibiotics with family members) of the antibiotics was common among the respondents (Table 3).

### Participants’ Attitude

Most of the participants had somehow been optimistic about antibiotics, and 13 participants believed that antibiotics had faster effect than any other medicines. Mostly everyone wanted to recover fast for daily activities, and use of antibiotics gave them extra strength, especially in infections. The majority of participants believed that many people got diarrhea after taking antibiotics and that they would rather take another medicine with the antibiotics if they got diarrhea.

Some of the interviewees had anger towards the local companies due to their low efficacy and preferred multinationally manufactured antibiotics. The participants had a favorable attitude toward the caring staff, as witnessed during the interview sessions, and the dealing person’s or pharmacy staff’s attitude at the pharmacy/drug outlet was important. Less than enough consumers who had a positive attitude were seen to be aware of antibiotic overuse (Table 4).

**TABLE 4:** Participants’ attitude towards antibiotics.

Theme 2: attitude towards antibiotics		
**Sub-themes**	**Categories**	**Quotations**
Attitude toward antibiotic use	In many cases, a positive attitude was seen toward obtaining quality antibiotics	I always preferred multinational and good quality pharmaceutical medicine companies’ antibiotics. I have experience with local companies’ antibiotics, it’s just a waste of money and has no effectiveness (respondent-B)
		I always ask about the pharmaceutical company name with its location and if the dealing person refer me local company antibiotics with a low price my trust of quality is down, then I refused to take an antibiotic (respondent-G)
		Whenever I need antibiotics, I go with physician prescriptions and ask for pharmacists to help but mostly pharmacists are absent at the pharmacy (respondent-H)
		I think the pharmacy/drug outlet staff is not concerned with the risk factors and misuse of antibiotics. The staff just looks at how much they will earn from the sale of antibiotics (respondent-A)
	Antibiotics can make a fast recovery	Antibiotics can heal infections and other conditions very quickly and that is the reason why I prefer antibiotics even for my sore throat as erythromycin works better than cold and flu medicines (respondent-I)
	To combat the diarrheal effect with metronidazole	Whenever I visit a pharmacy or drug store for antibiotics, I always request anaddition of metronidazole, which is the only prevention measure against diarrhea (respondent-G)
	The addition of paracetamol is mandatory to keep with antibiotics	I always self-add paracetamol with my antibiotics in case of fever with other conditions (respondent-C)

### Participants’ Practices

Participants’ practice regarding antibiotics was linked with their past illness or storage of antibiotics after feeling better. Some of the interviewees visited the physician for treatment but were directed to the specific pharmacy/drug outlet to buy the prescribed medications.

Three respondents always got advice from the staff at drug stores to obtain antibiotics. Due to the unavailability of physicians, other participants easily got antibiotics by just explaining the symptoms. Most of the participants stored the leaflet and empty packs of the antibiotics if they needed the same medicines next time for the same illness as the stored strips would help to get antibiotics without a prescription.

Five interviewees responded that “we open a leaflet inside the pack of antibiotics to read the instruction in Urdu, especially for the powder form and how to make a solution correctly” (respondent-O).

Eight participants agreed that they should follow the physicians’ instructions because the doctors know better. “I always listen to the physician for treatment processes and cross verify my prescription medicines with the physician once I obtain them from the pharmacy” (respondent-F).

#### Participants Believe in the Side Effects of Antibiotics

Ten respondents believed nausea and diarrhea occurred naturally due to any problem in the gastrointestinal system. The majority of the participants were unaware of the true cause and effects of incorrect antibiotic usage. Other interviewees explained that “because antibiotics are potent and might cause certain issues, it is recommended to take them with milk” (respondent-B) (Table 5).

**TABLE 5:** Participants’ practice toward antibiotics.

Theme 3: antibiotic practices		
**Sub-themes**	**Categories**	**Quotations**
Appropriate antibiotics consumption and healthcare professional consultation	Without consultation, antibiotics were used	The physician fee is too much for us, that is the reason we get antibiotics from drug stores without physicians and my relatives always help me in case they have antibiotics at home (respondent-C)
	The physician’s charges were outranged and his direction to a specific pharmacy	I had visited a physician for my recent illness, and he directed me to buy my medicines from the nearby pharmacy inside his clinic. I have tried to purchase the same antibiotics with the brand name but could not find in other locations (respondent-J)
	The pharmacist was new to consumers and didn’t ask about the medicines	Up till now, I did not even ask for the qualification from the medical/drug store staff and I do not know the pharmacist and the degree he holds (respondent-J)
	Lacking a pharmacist at the drug outlets/pharmacies	A pharmacist is a person who visits physicians to promote their products and I have never seen such a person at a medical store (respondent-I)
	The inappropriate practice of antibiotics	The pharmacy staff doesn’t guide us on the appropriate use of antibiotics and just put marks on the packs, sometimes it confused me to take my medications on empty stomach or after a meal (respondent-D)
		Instructions in Urdu are very easy to read and follow and I do read the instructions carefully (respondent-E)
	Antibiotics without prescription from a nearby place	I can get the medicines (antibiotics) without a prescription from nearby drug outlet (respondent-C)
	Reused previous stored antibiotics and sharing of the prescription as well	I always keep the strips, leaflet, and empty bottle of antibiotics at home (respondent-K)
	No worries about nonprescription antibiotics laws and the side effects	In our hometown, you can get all the types of medicines without a prescription easily, but in a city some pharmacies ask for a doctor’s prescription (respondent-E)

### Participants’ Recommendations to Improve the Antibiotic Use

All the respondents provided many recommendations to improve the antibiotic rational/reasonable use. Ten of the interviewees’ views were very positive to stop taking antibiotics from qualified healthcare professionals after physician consultation. Only a few interviewees had an answer such as “after completion of treatment, remaining antibiotics should be discarded on time” (respondent-L) (Table 6).

**TABLE 6:** Suggestions for the appropriate use of antibiotics.

Theme 4: how to improve appropriate (rational) use of antibiotics		
**Sub-themes**	**Categories**	**Quotations**
a) Suggestions to healthcare professionals	Have moral fear for nonprescription antibiotics and put all the responsibility on the healthcare professional	It is very wrong to take nonprescription antibiotics without consultation with a physician (respondent-A)
	Need community-based awareness programs	Many of us thought whenever we get sick taking medicines and antibiotics will recover you faster (respondent-B)
		Awareness is the main factor in the reduction of antibiotic resistance at the community level, and everyone should know the danger of misuse of antibiotics (respondent-E)
	The pharmacist must educate people	Once I met a pharmacist at the main city in his pharmacy, he gave me bits of advice on my antibiotics and counseled me on the proper use of antibiotics. I appreciate such efforts made by pharmacists (respondent-C)
	A prescriber must not prescribe unnecessary antibiotics	Doctors always prescribe unnecessary medicines, especially antibiotics and we take each dose on time as per the salesman or dispensing person’s instructions (respondent-D)
	Pharmacy staff must look twice at the antibiotic’s prescription rather than business perspectives	Most of the time, whenever I visit to buy required medicines all the staff have more focus on dispensing. No one cares to educate the patient on the prescribed antibiotics (respondent-A)
b) Suggestions for government policies	Strict regulations must be applied as soon as possible	Doctors along with other allied health sectors always call strike if the government tries to put new changes in the healthcare system, especially the drug act (respondent-D)
	Assured qualified pharmacist presence at drug outlet	The concerned health authorities must assure qualified and well-educated people at pharmacies as most of the medical/drug stores does not have such a person to guide a layman on their medicines (respondent-C)
	Doctors should be in contact with pharmacists	The linkage between pharmacies and physicians is more important as I had an issue with my prescription and I came the other day for the prescription confirmation from my doctor (respondent-I)
	Ban on private practice	Many public sector specialists are doing private clinical practices, the government must ban such practices and doctors must be available at their concerned hospitals (respondent-J)
	Antibiotics have separate regulations	Unqualified, without a pharmacy degree, people usually dispense antibiotics which is dangerous (respondent-A)
	Awareness is the need of the time	Awareness is the key to successful treatment and I have attended some sessions related to self-medications and now I educate people and discourage self-medications (respondent-F)

Eight participants, who belonged to the peripheral villages, responded that “the majority of the people in towns do not know about ABR.” Antibiotics and resistance to antibacterial agents should be made known to the general population through public awareness initiatives. The main reason for self-medication is education, and most of the population is deprived of basic education. The government must take serious action to improve awareness about antibiotic use and resistance at the community level with a very easy medium of local languages.

The doctor is responsible for making an accurate diagnosis and prescribing the proper antibiotics as per need. The majority of respondents had never heard of a pharmacist, and only two were aware of pharmacists and their involvement in medicine dispensing. The lack of pharmacists’ presence in the community pharmacies poses a threat to the spread of ABR. Most pharmacies, particularly in rural areas, are run by unqualified individuals. To limit antibiotic misuse, strict rules with severe fines implementation are required.

Antibiotic overuse can be curtailed by enforcing a policy that is implemented promptly. Three participants were well versed in the present provincial government regulations. Because the government failed to implement the new policies, five respondents did not believe in the government’s policies. Every time the government attempted to implement a new policy, healthcare professionals created roadblocks to prevent the implementation of new regulations and policies.

Every pharmacy should have a pharmacist on duty who can educate consumers on how to appropriately use antibiotics. Existing regulations and a national action plan on antimicrobial resistance in Pakistan might be used to combat ABR.

## Discussion

This study is the first of its kind to evaluate the consumers’ knowledge, attitudes, and practices toward antibiotic use and resistance in post-conflict areas of Pakistan. The AMR/ABR occurs mostly as a result of improper antibiotic use, and the current situation in Pakistan necessitates a great deal of attention. Antibiotics intake and behavior may be misinterpreted due to a lack of public understanding of self-medication with antibiotics (Alqarni and Abdulbari, 2019). Antibiotics are openly available without prescription in Pakistan, which is a unique situation to comprehend. Most people acquire and use medications based on their own needs, rather than seeing a doctor (Atif et al., 2019). ABR can be prevented by following the rules of right, i.e., using the right drugs (antibiotics) for the right patient. In healthcare settings ranging from hospitals to community pharmacies, the job of the qualified individual (pharmacist) is vital.

Our findings are in concordance with the results of other studies. According to many research reports conducted in developed and emerging EU nations, antibiotics are being misused by individuals for ordinary diseases (Väänänen et al., 2006; Kotwani et al., 2021). Antibiotic knowledge is undoubtedly complex in many aspects, yet we endeavored to give an antibiotic-related booklet to the participants in the hopes that it would have a positive impact on antibiotic attitudes and behaviors. Education is the most important factor for inappropriate antibiotic usage in a specific community, and ABR might be minimized to some extent with increased understanding and education, according to prior research (Majumder et al., 2020). According to Khan et al., many factors, including sociological contextual factors and political and economic concerns, can play a stronger role in the fight against ABR (Krockow et al., 2019). Lack of knowledge and awareness about antibiotics is frequently the cause of antibiotic overuse (Aponte-González et al., 2019). A positive mindset, suitable information, and practices are all important factors in the proper and limited use of antibiotics (Napolitano et al., 2019). The AMR/ABR, which develops as a result of antibiotic abuse, is an issue that is currently receiving a lot of attention in Pakistan. Due to a lack of public information, attitude, and practices, antibiotic intake and behaviors may be misconstrued (Alqarni and Abdulbari, 2019). In Pakistan, antibiotics are readily available, which is a unique scenario to grasp (Atif et al., 2019). Consumers in our survey complained that doctors or pharmacists did not freely offer comprehensive information (Lum et al., 2017). In both the developed and developing worlds, ABR is a major concern (Lum et al., 2017).

Convenience and cost savings have often been found in studies of merging nations such as China and several European countries (Melchiorre et al., 2013; Nawafleh et al., 2016; Hayat et al., 2019). Aslam et al. identified the most relevant determinants in LMICs for self-medication with antibiotics, concluding that education and mass media actions to raise public knowledge of the risks and side effects of self-medication were the most essential variables (Aslam et al., 2020). Contextual and complete research on characteristics influencing nonprescribed antibiotic usage, particularly in LMICs, is essential to effectively address antibiotic use and control the problem of ABR (Torres et al., 2019).

Even though the consumer sample was uneducated, there were variations in understanding, sensitivity, and concerns about antibiotics and ABR. Some of the inaccuracies, according to another study, suggest that clear communications from health experts, public health initiatives, and the media are essential all of the time (Lum et al., 2017). The individuals seemed to find antibiotics to be a source of hope. Antibiotics have a faster effect than any other treatment, according to more than ten participants, which is why they are not usually prescribed (Jacobs et al., 2019). According to another study, the majority of participants believed that many people get diarrhea after taking antibiotics and that if they received diarrhea, they would prefer to take another antibiotic-containing drug (Eibl et al., 2021). Other studies found that participants’ usage of antibiotics was linked to previous illness or the storage of medications after they had recovered, when it comes to the usage of antibiotics, malpractices made numerous errors. As observed in both the current and previous studies, the majority of participants’ activities were linked to the preservation of empty strips, packs of antibiotics for the next time usage of the same drugs for the same disease. The French Government supported an antibiotic campaign from 2002 to 2007 to reduce antibiotic use by a variety of actors, including the general public. This campaign proved successful in France, with a significant decrease in antibiotic use (Sabuncu et al., 2009). Such initiatives and movements are required at the national level in Pakistan.

Furthermore, to avoid antibiotic scarcity, government agencies must develop strict regulations. Our findings demonstrate that most consumers are uninformed of existing antibiotics norms, laws, and national action plans, which are also misunderstood. According to a Jordanian study that made similar recommendations, controlling unnecessary antibiotics should be a key priority for physicians, pharmacists, and other regulatory organizations (Khasawneh et al., 2021). The matter must be brought to the attention of regulatory authorities, and better policy development based on our findings would be advantageous. For the culture to change, both the doctor and the pharmacist must collaborate professionally.

The lack of rigorous government laws and regulations was observed in our findings, as the interviewees were indifferent to the restrictions. ABR rates may be reduced because of community-based antimicrobial education efforts. According to studies, Pakistani physicians are supportive of antimicrobial stewardship efforts. The main problem is that rules and procedures differ from state to state (Hashmi et al., 2021; Jabeen et al., 2021).

This study is accompanied by a few limitations: first, it was conducted in post-conflict regions where the people had been through multiple military operations, and the findings may not be generalized to the other parts of the country. Second, many people have stories to tell, notably about the destruction of healthcare institutions and infrastructure in the scenic valley famed for tourism. Third, due to the cultural values of the data collection sites, fewer females took part in our study at the outset. A brief explanation of the laws and regulations, as well as Pakistan’s national action plan on AMR, was provided before going on to note the suggestions on the proper use of antibiotics. Fourth, the outcome could have been influenced by consumer counsel. Furthermore, the study was only conducted in one district, but it might be expanded to other parts of Pakistan, using focus groups and interventional qualitative research. Following the in-depth interviews with the research participants, a brief intervention was conducted to present the relevant and critical information on antibiotic use, ABR, and measures to improve antibiotic consumption.

## Conclusion

This study underscored the poor knowledge of residents from the conflicted regions regarding the appropriate use of antibiotics and ABR. Most of the study participants were aware of the term “antibiotic” without proper knowledge of antibiotic usage. However, the respondents could not grasp the concept of resistance completely. A somehow positive attitude was seen among the consumers, but the absence of a prescription, self-medication, antibiotic storage at home, and medicine sharing was found as significant contributors to incorrect antibiotic use. Malpractices in the use of antibiotics were common among the participants. It had also been observed that the leftover antibiotics were saved for future use after the therapy was completed. The absence of a qualified person, charges related to physicians visits, unnecessary prescriptions, and timely implementation of existing laws were the main areas suggested by the participants. Lack of awareness, remote health services, a shortage of pharmacists, and unregulated drug access had all been linked to insufficient antibiotic use. Further research is needed to address the issue of ABR in Pakistan’s post-conflict areas.

## Data Availability

The raw data supporting the conclusions of this article will be made available by the authors upon a reasonable request.
