# Supplementary material for: Evaluation of Consumers Perspective on the Consumption of Antibiotics, Antibiotic Resistance, and Recommendations to Improve the Rational use of Antibiotics: An Exploratory Qualitative Study From Post-Conflicted Region of Pakistan
Source: Front Pharmacol. 2022 May 18;13:881243. doi: 10.3389/fphar.2022.881243 (PMC9159815; doi:10.3389/fphar.2022.881243)
Supplement: Supplementary file 1 [file DataSheet1.PDF]

INTERVIEW GUIDE FOR CONSUMERS

Exploring the determinants of consumers perspectives on antibiotics use, and antibiotic resistance at community pharmacies: A exploratory qualitative study in post-conflict areas

PARTICIPANT INFORMATION SHEET -شرکاء کے لیے معلوماتی شیٹ

آپ کو ایک تحقیقی مطالعہ میں شرکت کے لیے مدعو کیا جا رہا ہے جس کا عنوان ہے "اینٹی بائیوٹک کے استعمال اور اینٹی بائیوٹک مزاحمت کے بارے میں صارفین کے علم ، رویہ اور طریقوں کے بارے میں :پاکستان کے جنگ کے بعد کے علاقوں سے تحقیقاتی مطالعہ۔

Purpose of study:

This study aims to assess the understanding of the consumer's knowledge, attitude, and practices towards antibiotics use and antibiotic resistance following semi-structured interviews.

اس مطالعے کا مقصد نیم ساختہ انٹرویوز کے بعد اینٹی بائیوٹکس کے استعمال اور اینٹی بائیوٹک مزاحمت کے بارے میں صارفین کے علم ، رویہ اور طریقوں کی تفہیم کا جائزہ لینا ہے۔

Your Participation:

Your participation in this study is entirely voluntary and you can withdraw at any time. We believe there are no known risks associated with this research study. To the best of our ability, your answers in this study will remain confidential. We assure you that all data collected will be anonymous. All data will be stored electronically on password-protected media for a maximum of three years. All data collection, storage, and processing will comply with the principles of Data Protection.

اس مطالعے میں آپ کی شرکت مکمل طور پر رضاکارانہ ہے اور آپ کسی بھی وقت دستبردار ہو سکتے ہیں۔ ہمیں یقین ہے کہ اس تحقیقی مطالعے سے وابستہ کوئی معروف خطرات نہیں ہیں۔

B. INFORMED CONSENT

You will be asked to sign an informed consent stating that you understand the nature of the study and what is required from you in the study. Participation in this study is voluntary and will not affect your privacy. You are free at any time to change your mind and withdraw from the study without needing to justify your decision.

* By completing this survey please click AGREE to indicate that:

• You are a resident of swat

• Your prescription includes antibiotics

• You are willing to participate in the present study

• You have read the participant information sheet associated with this study

آپ سے کہا جاتا ہے کہ ایک باخبر رضامندی پر دستخط کریں جس میں کہا گیا ہے کہ آپ مطالعے کی نوعیت کو سمجھتے ہیں اور مطالعے میں آپ سے کیا ضروری ہے۔ اس مطالعے میں شرکت رضاکارانہ ہے اور آپ کی رازداری کو متاثر نہیں کرے گی۔ آپ کسی بھی وقت اپنے ذہن کو تبدیل کرنے اور اپنے فیصلے کو جائز قرار دینے کے بغیر مطالعے سے دستبردار ہونے کے لیے آزاد ہیں۔

Sign (optional) ____________

Theme 1: Knowledge

1. What do you understand by the term ‘antibiotic? and where do you hear the term before?

If yes, please give your opinion/definition of antibiotics

a). Antibiotics (General) knowledge

Antibiotics are effective in Conditions/diseases like;

Conditions/diseases like;

- Diarrhea
- Skin infections
- Sore throat
- Cold n flu
- Malaria
- Fever
- Headache or body aches

b). Knowledge of Antibiotics Resistance

i. What do you understand by the term ‘antibiotic resistance’?

ii. When antibiotic resistance developed? Misuse of antibiotics are linked with antibiotic resistance?

Theme 2: Attitude

a). Attitude towards antibiotics use

i. *Antibiotics can make a fast recovery from any illness? Or specific to infections?*
ii. *To combat the diarrheal effect with metronidazole is a a good fit?*
iii. *Storage of unnecessary antibiotics at home?*

Theme 3: Practices

a). Health care professional’s consultation is very important for a patient? Especially pharmacist and physician

i. *Pharmacist role at the pharmacy*
ii. *Physicians' charges and directions for antibiotics*
iii. *Pharmacist at the drug outlets/pharmacies*
iv. *Antibiotics without prescription*
v. *Use of previously stored antibiotics and sharing with others*

vi. Theme 4: How to improve appropriate (rational) use of antibiotics

a). Suggestions to Healthcare professionals

i. *Antibiotics are easily available? Physicians have an empiric prescription for the antibiotics.*
ii. *Awareness programs*
iii. *Pharmacist counseling*

b). Suggestions for Government policies

i. *Current regulations*
ii. *Qualified pharmacist presence*
iii. *Doctors and pharmacist's communication*
iv. *Antibiotics regulations and implementations*
